# *WUSCHEL-Related Homeobox* (*WOX*) Gene Family in Quinoa (*Chenopodium quinoa*): Genome‐Wide Identification and In Silico Characterization

**DOI:** 10.1155/ijog/7924847

**Published:** 2025-09-15

**Authors:** Bahlanes Bakhtari, Elnaz Zamani

**Affiliations:** ^1^ Department of Plant Production and Genetics, School of Agriculture, Shiraz University, Shiraz, Iran, shirazu.ac.ir

**Keywords:** protein–protein interaction, purifying selection, quinoa, *WOX* gene family

## Abstract

Plant‐specific transcription factors known as WUSCHEL‐related homeobox (WOX) proteins are crucial for regulating plant development and responses to stress. This study represents the first thorough characterization of the *WOX* gene family in quinoa (*Chenopodium quinoa*, *CqWOX*s). In total, we identified 13 probable *CqWOX* genes, which were categorized into three main subgroups (ancient subgroup, intermediate subgroup, and WUS subgroup) based on phylogenetic analysis. Synteny analysis revealed 12 *CqWOX* genes as orthologs of *WOX* genes in *Beta vulgaris*, *Amaranthus hypochondriacus*, and *Spinacia oleracea*, while 10 orthologs were found in *Arabidopsis thaliana*. Five segmentally duplicated *WOX* gene pairs were identified in the quinoa genome, all of which have undergone purifying selection, as indicated by their Ka/Ks values being less than one. Additionally, the 2.0 kb promoter regions of *CqWOX*s were found to harbor various *cis*‐acting elements related to hormone‐responsiveness, stress‐responsiveness, growth and development, and light‐responsiveness elements. The protein–protein interaction network established included 6 Arabidopsis WOX proteins and 10 other notable proteins that showed strong interactions with WOX proteins, comprising 16 nodes in total. Transcriptome analysis demonstrated that *CqWOX* genes in quinoa exhibit both tissue‐specific and salt‐responsive expressions. Several genes were repressed in salt bladders, while others showed increased expression, suggesting their potential roles in stress adaptation. Expression profiles differed in leaf and root tissues under salt stress conditions. This study suggested that *CqWOX* genes exhibit unique characteristics that may facilitate further investigation in future research.

## 1. Introduction

Homeobox (HB) proteins are part of a superfamily of transcription factors (TFs) that are essential in regulating morphogenesis and developmental processes in eukaryotes. These proteins possess a highly conserved HB domain, typically containing 60–66 amino acids, which mainly functions as a DNA‐binding domain that can either activate or repress gene expression [1]. In plants, HB proteins are classified into 14 distinct sets based on conserved motifs, such as HD‐ZIP I–IV, DDT, BEL, LD, KNOX, PLINC, NDX, PINTOX, PHD, SAWADEE, and *WUSCHEL-related homeobox* (*WOX*) [2].

In higher plants, the *WOX* genes code for a family of plant‐specific TFs that have been identified and described in Arabidopsis (*Arabidopsis thaliana*), maize (*Zea mays*), rice (*Oryza sativa*), and various other plant species [3, 4]. Based on phylogenetic evolutionary relationships, members of the *WOX* family are divided into three distinct subgroups. The WUSCHEL (WUS)/modern subgroup is exclusive to seed plants, the intermediate subgroup is found in vascular plants, and the ancient subgroup is present in both nonvascular and vascular plants [5–7].

In Arabidopsis, regarded as a model plant, 15 *WOX* genes have been meticulously identified. The WUS subgroup, compared to the intermediate and ancient subgroups, consists of a larger number of members and has been subject to more extensive studies. The first *WOX* gene discovered in Arabidopsis was WUS, recognized as a vital conserved regulator necessary for maintaining the stem apical meristem and for the development of floral organs [8].

Previous studies indicate that *WOX* family genes function as multifunctional TFs involved in various aspects of plant growth and development throughout the full life cycle of the plant. Their roles range from maintaining meristems to embryonic patterning, as well as from the development of lateral organs to tolerance against abiotic stress [9, 10].

*WOX* genes are found across a wide range of plant species, from basic organisms like green algae to more advanced plants such as Arabidopsis, with varying numbers in different species [11]. Throughout plant evolution, the *WOX* gene family has gradually grown. For instance, unicellular green algae like *Ostreococcus lucimarinus* and *Ostreococcus tauri* have only one *WOX* gene, while *Physcomitrella patens* has three [6], *Selaginella tamariscina* features six [2], *Medicago sativa* contains 14 [12], *A. thaliana* includes 15 [13], *Cucumis sativus* possesses 11 [14], *Helianthus annuus* contains 10 [15], and *Z. mays* has 21 *WOX* genes [4]. Previous studies have emphasized the vital functions of *WOX* family members in embryonic development, stem cell maintenance, and organ formation [4, 11]. Despite quinoa (*Chenopodium quinoa*) being recognized as a highly nutritious traditional food crop, there has been no research on the *WOX* gene family in this crop. As sedentary organisms, crops regularly face tough environmental conditions, where abiotic stress significantly affects their growth rate, cellular metabolism, and overall productivity [16]. Notably, quinoa shows impressive tolerance to several abiotic stresses, including cold, salinity, drought, and nutrient‐poor soils. Its ability to thrive in marginal lands unsuitable for staple crops like rice and maize highlights its potential as a nutritious food source. As a facultative halophyte, quinoa is highly resilient to drought and low temperatures. However, it remains an underutilized crop with limited breeding programs despite its agricultural potential. To boost global quinoa production, breeding programs should aim to enhance key agronomic traits. The main obstacles in cultivating quinoa outside its native areas include its relatively low yield, high sensitivity to changes in photoperiod, and the absence of focused breeding efforts tailored to specific environmental conditions [17].

The genome‐wide identification of the *WOX* gene family in quinoa, along with the exploration of their functions, molecular pathways, and target genes, can provide valuable insights into the molecular mechanisms underlying quinoa development and stress reactions. In this study, the *WOX* family members were discovered within the quinoa genome, followed by an extensive bioinformatics analysis to explore their phylogenetic relationships, conserved protein domains, gene structure, chromosomal distribution, *cis*‐regulatory elements (CREs), and duplication events. This study presents the first comprehensive identification and characterization of 13 *CqWOX* genes in quinoa, providing new insights into the evolutionary dynamics of *WOX* genes within the Amaranthaceae family. These discoveries lay a fundamental theoretical foundation for further functional studies of these genes and contribute to the broader understanding of the *WOX* gene family across different plant species.

## 2. Materials and Methods

### 2.1. Identification and Properties of CqWOX Proteins

The WOX protein sequences from *A. thaliana*, *O. sativa*, *Beta vulgaris*, *Amaranthus hypochondriacus*, and *Spinacia oleracea* were obtained from the Plant TF Database (https://planttfdb.gao-lab.org/) [18]. To perform a genome‐wide identification of WOX family proteins in quinoa, WOX sequences from *A. thaliana* were used as query sequences for BLASTP explorations against the Phytozome V13 (https://phytozome-next.jgi.doe.gov/) with default settings and *E* − value < 1e^−10^. Redundant sequences were excluded using the Expasy online platform (https://web.expasy.org/decrease_redundancy/) with elimination threshold: sequence similarity > 90% [19]. Subsequently, the unique protein sequences remaining were then analyzed to verify the presence of the homeodomain (PF00046), a defining trait of WOX proteins, through the simple modular architecture research tool (SMART) (http://smart.embl-heidelberg.de/) [20]. To identify the DNA‐binding helix‐loop‐helix‐turn‐helix domain, multiple sequence alignment was performed using the ClustalX 2.1 tool [21].

The theoretical isoelectric point (pI) and molecular weight (MW) of the predicted CqWOX proteins were determined using the ProtParam tool (http://web.expasy.org/protparam/) [22]. Additionally, the subcellular localization of CqWOX proteins was forecasted utilizing the Bologna Unified Subcellular Component Annotator (BUSCA) tool (https://busca.biocomp.unibo.it/) [23].

### 2.2. Molecular Modeling of CqWOX Proteins

The secondary structure of CqWOX proteins was predicted using the SOPMA online tool [24], which identifies structural elements such as *α*‐helices, random coils, *β*‐turns, and extended strands. Additionally, the three‐dimensional structures of the CqWOX proteins were modeled using the SWISS‐MODEL server (https://swissmodel.expasy.org/) [25] to predict domain organization and overall structural features.

### 2.3. Chromosomal Location, Phylogenetic Analysis, and Synteny Analysis of *WOX* Genes

The chromosomal locations of *WOX* TFs found in quinoa were established using the OMA browser (https://omabrowser.org/oma/genome/CHEQI/info/). The physical mapping of the identified *CqWOX* genes was carried out using MG2C V2.1 (http://mg2c.iask.in/mg2c_v2.1/) [26]. To create phylogenetic trees for the WOX family, which includes proteins from quinoa, *A. thaliana*, *O. sativa*, *B. vulgaris*, *A. hypochondriacus*, and *S. oleracea*, multiple sequence alignments were performed using ClustalX 2.1 [21], followed by tree construction based on the neighbor‐joining (NJ) method with 2000 bootstrap replicates. The aligned protein sequences were then used to create a phylogenetic tree via the iTOL web tool (https://itol.embl.de/) [27]. Additionally, to investigate collinearity and syntenic relationships between quinoa and four crop species (*A. thaliana*, *B. vulgaris*, *S. oleracea*, and *A. hypochondriacus*), whole‐genome sequences and their corresponding genome annotation files were analyzed using MCScanX within the TBtools software (V2.057) [28, 29].

### 2.4. Conserved Motifs, Gene Duplication, and Ka/Ks Analysis

The conserved motifs within CqWOX proteins were discovered using the MEME Suite (https://meme-suite.org/meme/tools/meme) [30] with the following parameters: (1) Each sequence could contain either zero or one instance of a specific motif, (2) a maximum of 15 motifs was permitted, (3) the optimal width of motifs was set between 6 and 50 amino acids, and (4) only motifs with *E*‐values below 0.05 were viewed as significant. Next, the biosequence structure illustrator tool in TBtools software (V2.057) [28, 29] was utilized to visualize the conserved domains, exon–intron structures, motifs, and CREs of the *CqWOX* genes.

The “Simple Ka/Ks Calculator” in TBtools [28, 29] was used to compute the nonsynonymous (Ka) and synonymous (Ks) substitution rates for gene duplication pairs. Selection pressure was interpreted based on Ka/Ks values: Ka/Ks < 1 indicated purifying selection, Ka/Ks = 1 suggested neutral selection, and Ka/Ks > 1 implied positive selection [31]. The divergence time of *CqWOX* gene pairs, measured in million years ago (MYA), was estimated using the formula *T* = Ks/2*λ* (where *λ* = 6.5 × 10^−9^), with *λ* representing the synonymous substitution rate per year [32].

### 2.5. Promoter Analysis, Construction of Protein–Protein Interaction (PPI) Network, and Gene Ontology (GO)

The promoter region was designated as the 2000 bp sequence upstream of the transcription start site of *CqWOX* genes. Predictions of *cis*‐acting elements within these promoter regions were made with the PlantCARE (http://bioinformatics.psb.ugent.be/webtools/plantcare/html/) database [33]. Additionally, the STRING software (V12.0; https://string-db.org/) [34] was utilized to predict PPI networks for CqWOX sequences by comparing them with their homologous proteins from *A. thaliana*. To gain deeper insights into the functions of *CqWOX* genes, GO annotation analysis was conducted using the ShinyGO 0.82 tool [35].

### 2.6. RNA‐Seq Data Analysis

To identify *CqWOX* genes with differential expression, publicly available RNA‐Seq datasets were analyzed, including samples derived from quinoa salt bladders, leaves without bladders, and whole leaves (NCBI BioProject: PRJEB22942). Raw reads were quality‐filtered by trimming sequences with a minimum quality score below 0.05 and discarding reads containing more than two ambiguous nucleotides. Adapter sequences and reads shorter than 15 nucleotides were also removed. The high‐quality reads were mapped to the quinoa reference genome (GCA_001742885.1) retrieved from NCBI. Gene expression levels were quantified using the TPM (transcripts per million) method, and normalization was performed using the quantile method described by Van den Broeck et al. [36]. Differentially expressed genes (DEGs) were identified using Baggerley’s test as implemented in CLC Genomics Workbench 21, applying a false discovery rate (FDR) threshold of 0.05 and a |fold change| ≥ 1.5. In addition, to assess *CqWOX* gene expression in response to salinity stress, we analyzed RNA‐Seq data from NCBI BioProject PRJNA605324, previously published by Vita et al. [37].

## 3. Results

### 3.1. Identification and Properties of CqWOX Proteins

A total of 13 *WOX*‐encoding genes were identified in the quinoa genome. These genes were named based on their chromosomal location [38] as *CqWOX1* to *CqWOX11* and *CqWUS1* and *CqWUS2*, respectively (Table 1). The length of the proteins produced by the quinoa WOX gene family ranged from 159 to 676 amino acid residues (Table 1). The MWs of these proteins varied from 18.5 (CqWOX1) to 76.72 kDa (CqWOX10), while their pIs spanned from 5.15 (CqWUS1) to 9.47 (CqWOX4). Predictive subcellular localization analysis indicated that all CqWOX proteins, except CqWOX2, are localized in the nucleus (Table 1).

**Table 1:** Physical properties of *CqWOX* gene family.

**Gene ID**	**Name**	**Chr_id**	**CDS length (bp)**	**Number of amino acids (AA)**	**Molecular weight (kDa)**	**Isoelectric pi (pI)**	**Subcellular localization (SL)**
AUR62017610	*CqWOX1*	Chr00	480	159	18.505	8.62	Nucleus
AUR62014909	*CqWOX2*	Chr01	1035	344	37.177	6.73	Chloroplast
AUR62031114	*CqWOX3*	Chr02	1056	351	37.957	6.94	Nucleus
AUR62031363	*CqWOX4*	Chr03	702	233	26.734	9.47	Nucleus
AUR62012213	*CqWOX5*	Chr03	585	194	22.247	8.5	Nucleus
AUR62022846	*CqWOX6*	Chr04	537	178	20.462	8.58	Nucleus
AUR62001809	*CqWOX7*	Chr07	1893	630	71.231	5.53	Nucleus
AUR62002048	*CqWOX8*	Chr07	603	200	22.682	5.6	Nucleus
AUR62003747	*CqWOX9*	Chr09	861	286	31.821	5.27	Nucleus
AUR62009597	*CqWOX10*	Chr18	2031	676	76.729	6.02	Nucleus
AUR62006532	*CqWOX11*	Chr18	1509	502	56.650	6.1	Nucleus
AUR62003373	*CqWUS1*	Chr06	798	265	29.426	5.15	Nucleus
AUR62029457	*CqWUS2*	Chr15	807	268	28.487	5.34	Nucleus

**Figure 1:**
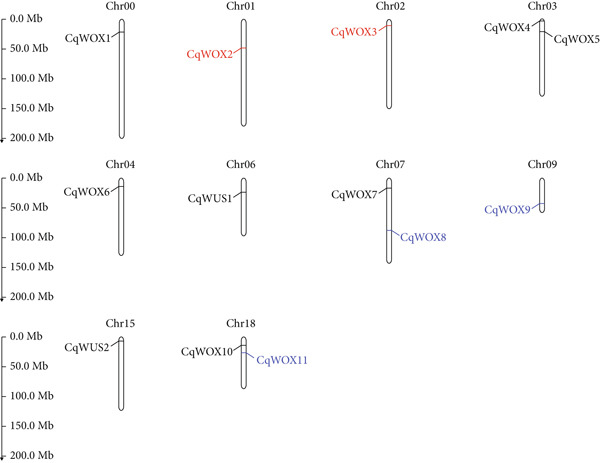
Distribution of *CqWOX* genes in quinoa chromosomes. The ancient subgroup genes are represented in blue, the intermediate subgroup genes are in red, and the WUS subgroup genes are in black.

Chromosomal localization analysis (Figure 1) demonstrated that 12 *CqWOX* genes were spread across nine chromosomes, with one gene remaining unmapped, assigned to chromosome zero [39]. Notably, no *WOX* family members were found on Chromosomes 5, 8, 9–14, 16, and 17. Each of Chromosomes 0, 1, 2, 4, 6, 9, and 15 contained a single *WOX* gene, while Chromosomes 3, 7, and 18 each had two *WOX* genes (Figure 1).

### 3.2. Phylogenetic and Synteny Analysis of *CqWOX* Genes

**Figure 2:**
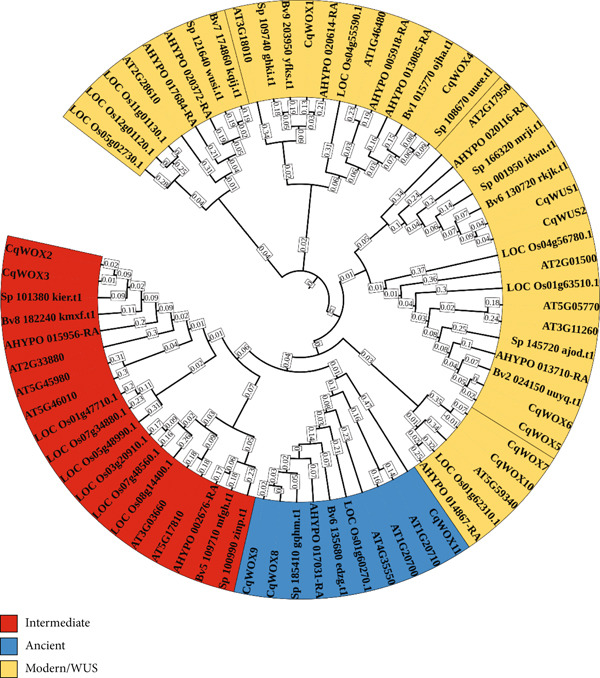
Phylogenetic analysis of *WOX* TF family across *Chenopodium quinoa*, *Oryza sativa*, *Arabidopsis thaliana*, *Amaranthus hypochondriacus*, *Beta vulgaris*, and *Spinacia oleracea*. The phylogenetic tree contains 13 CqWOXs, 16 AtWOXs, 14 OsWOXs, 9 SpWOXs, 11 AhWOXs, and 8 BvWOXs, categorized into three subgroups represented by different colors. The ancient subgroup genes are represented in blue, the intermediate subgroup genes are in red, and the WUS subgroup genes are in orange.

To gain a deeper understanding of the functional domains within the *CqWOX* gene family and to explore the evolutionary relationships among typical monocots (*O. sativa*), dicots (*A. thaliana*), and three plant species from the Amaranthaceae family (*B. vulgaris*, *A. hypochondriacus*, and *S. oleracea*), a phylogenetic analysis of the *WOX* gene family was conducted (Figure 2). In total, 71 WOX proteins were analyzed, including 16 from *A. thaliana*, 8 from *B. vulgaris*, 9 from *S. oleracea*, 11 from *A. hypochondriacus*, 14 from *O. sativa*, and 13 from *C. quinoa* (Figure 2). The resulting phylogenetic tree clearly grouped these WOX proteins into three major subgroups: the ancient subgroup, the intermediate subgroup, and the WUS subgroup.

The results indicated that the WUS, comprising eight CqWOX members, was the largest subgroup, surpassing the total number of proteins in both the ancient subgroup (three members) and the intermediate subgroup (two members). Additionally, the close phylogenetic relationship between *C. quinoa* WOX proteins and their homologs in the three Amaranthaceae species points to a significant level of evolutionary conservation among WOX TFs within this plant family.

**Figure 3:**
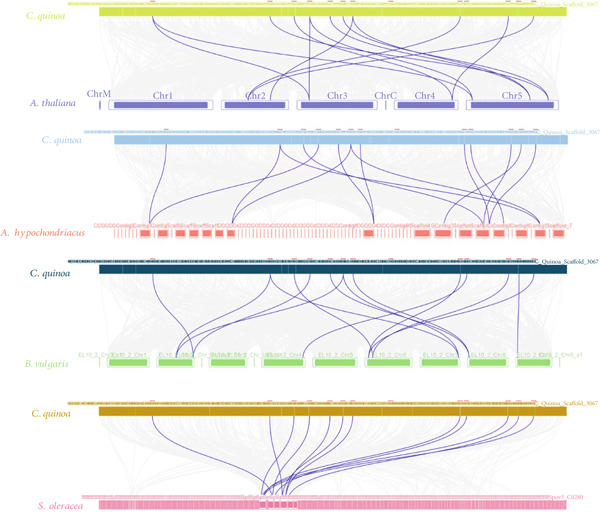
Syntenic relationships between the homologous *CqWOX* genes in quinoa and other species.

Synteny analysis is a fundamental approach in comparative genomics, essential for assessing the molecular evolutionary relationships among different species [17]. A comparative synteny analysis was conducted to uncover orthologous relationships of *WOX* genes between *C. quinoa* and three other Amaranthaceae species—*B. vulgaris*, *A. hypochondriacus*, and *S. oleracea*—as well as the model species *A. thaliana* (Figure 3).

According to the results, the *CqWOX* genes have 12 orthologs with *WOX* genes in three other Amaranthaceae species and 10 orthologs with *WOX* genes in *A. thaliana* (File S1). Interestingly, specific *CqWOX* genes, such as *CqWOX7* and *CqWOX10*, showed orthologous relationships with at least two gene pairs, especially with *WOX* genes from *B. vulgaris* and *A. hypochondriacus*. This indicates that these genes may have played significant roles in the evolutionary path of the *CqWOX* gene family. Moreover, it is noteworthy that only the *CqWOX4* gene was not found in the other species analyzed (File S1), suggesting that the majority of *WOX* genes have been conserved throughout evolution due to their significance.

### 3.3. Analysis of Gene Structure and Conserved Motifs of *CqWOX*s

**Figure 4:**
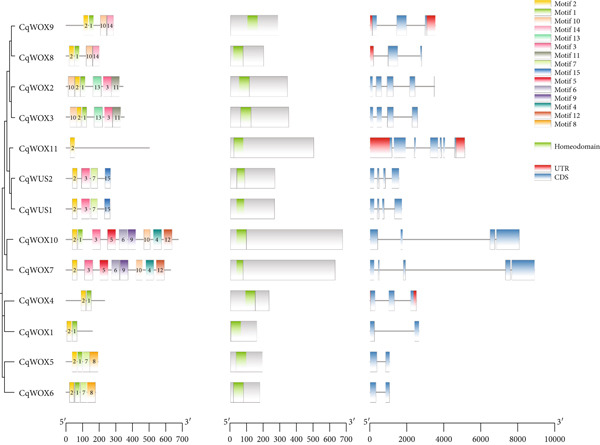
Phylogenetic relationships, conserved motifs, positions of domains, and positions of exons and introns in *CqWOX* genes.

To further investigate the evolutionary characteristics of *CqWOX* genes, we analyzed the exon–intron structures of the *CqWOX* gene family in quinoa by comparing coding sequences (CDS) to their corresponding genomic sequences. The findings showed a variety of exon–intron structures among *CqWOX* genes. Structural analysis revealed a range in the number of exons, from two (in *CqWOX1*, *CqWOX5*, *CqWOX6*, and *CqWOX8*) to seven (in *CqWOX11*). Of the *CqWOX* genes analyzed, 30.7% contained two exons, while another 30.7% had four exons, with only *CqWOX11* having seven exons. A strong correlation was observed between the exon–intron structures and the phylogenetic relationships of *CqWOX* genes (Figure 4).

**Figure 5:**
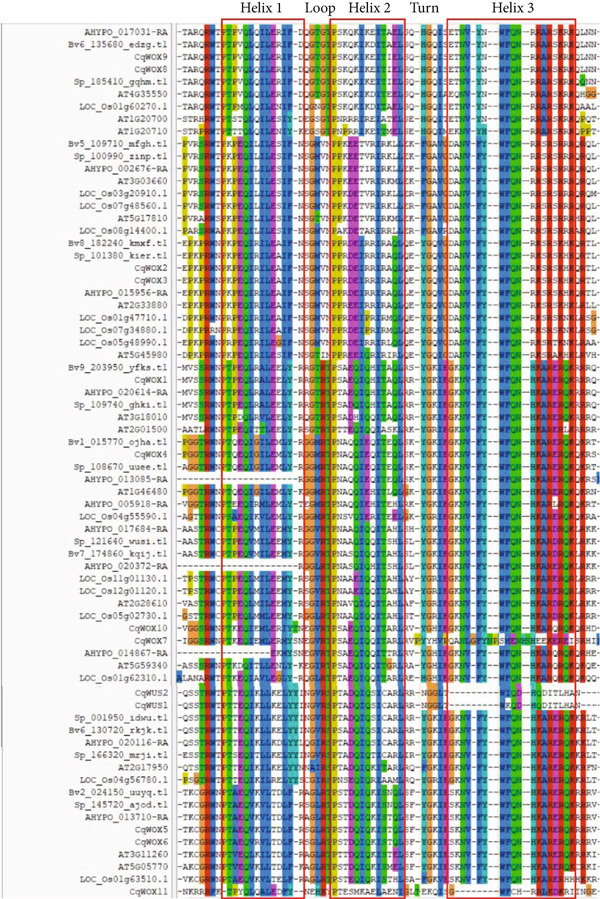
Multiple sequence alignment of WOX homeodomain of *Chenopodium quinoa*, *Oryza sativa*, *Arabidopsis thaliana*, *Amaranthus hypochondriacus*, *Beta vulgaris*, and *Spinacia oleracea*.

In Arabidopsis, three functional domains—the WUS‐box, the EAR‐like motif, and the acidic region—have been recognized within members of the WUS subgroup, which are critical for protein function [40]. In quinoa, the WUS‐box (TLXLFP) was found in several WUS subgroup members, including CqWOX1, CqWOX4, CqWOX5, CqWOX6, CqWUS1, and CqWUS2. Additionally, the EAR‐like motif (LXLXL, Motif 8) was specifically associated with putative *WUS* genes (File S2, Figure 4). Interestingly, two TFs in quinoa, CqWUS1 and CqWUS2, included the LELXL motif (Motif 15) (Figure 4). Motif analysis revealed that all CqWOX proteins contained Motif 2, while Motifs 11 and 13 were exclusive to the intermediate subgroup. Moreover, Motif 1 was broadly shared across most protein sequences. The detection of conserved motifs within the same phylogenetic subgroup suggests a degree of functional conservation, as proteins within a particular subgroup exhibited similar motif compositions beyond the conserved regions. Multiple sequence alignment revealed that all CqWOX proteins possess a conserved DNA‐binding helix‐loop‐helix‐turn‐helix domain (Figure 5).

### 3.4. Molecular Modeling of CqWOX Proteins

**Figure 6:**
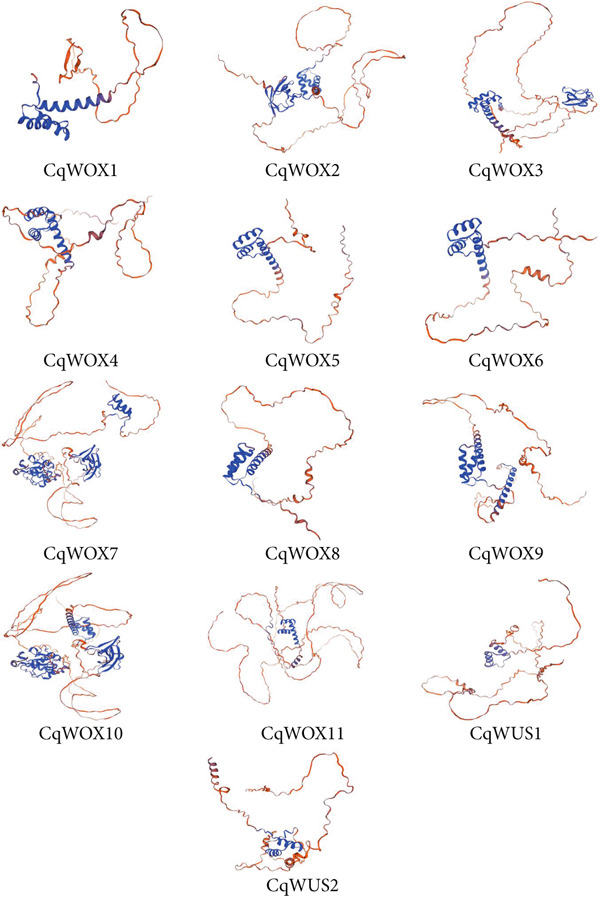
Prediction of the tertiary structure of quinoa WOX family proteins.

To gain insight into the structural characteristics of CqWOX proteins, homology‐based modeling was performed using AtWOX proteins as templates in the SWISS‐MODEL platform. The analysis revealed that, despite variations in overall length and morphology among the 13 CqWOX proteins, all possessed a conserved helix‐loop‐helix homeodomain—a hallmark feature of plant WOX proteins (Figure 6). This observation was consistent with the results of the sequence alignment analysis (Figure 5). Structural predictions further indicated that these proteins were predominantly composed of random coils, ranging from 60.38% in CqWOX1 to 78.11% in CqWUS1, while alpha‐helical regions varied from 9.88% in CqWOX2 to 25.26% in CqWOX5 (Table 2).

**Table 2:** Secondary structure in CqWOX proteins.

**Protein**	**Alpha helix**	**Extended strand**	**Beta turn**	**Random coil**
CqWOX1	25.16%	9.43%	5.03%	60.38%
CqWOX2	9.88%	10.17%	5.52%	74.42%
CqWOX3	9.97%	9.97%	3.13%	76.92%
CqWOX4	17.60%	5.58%	2.58%	74.25%
CqWOX5	25.26%	7.73%	2.58%	64.43%
CqWOX6	24.72%	3.93%	2.81%	68.54%
CqWOX7	19.84%	11.43%	2.22%	66.51%
CqWOX8	17.50%	4.00%	2.50%	76.00%
CqWOX9	23.08%	4.20%	1.75%	70.98%
CqWOX10	21.60%	8.73%	3.85%	65.83%
CqWOX11	23.51%	1.79%	1.39%	73.31%
CqWUS1	17.36%	2.64%	1.89%	78.11%
CqWUS2	19.40%	1.87%	1.87%	76.87%

### 3.5. Gene Duplication Events of the Quinoa *WOX* Family

**Figure 7:**
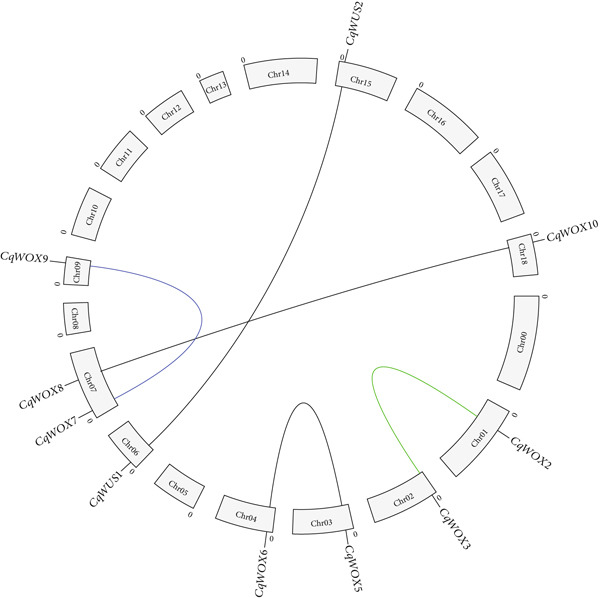
Genomic distribution of the *CqWOX* genes and gene homology analysis in quinoa.

Gene duplication plays a critical role in species evolution by expanding the number of functional genes [12]. A duplicated gene pair located on different chromosomes is defined as a segmentally duplicated gene pair. In contrast, a nearby pair on the same chromosome is categorized as a tandemly duplicated gene pair [41]. In the quinoa genome, five pairs of segmentally duplicated *WOX* genes have been identified: *CqWOX9*/*CqWOX8*, *CqWOX2*/*CqWOX3*, *CqWOX5*/*CqWOX6*, *CqWOX10*/*CqWOX7*, and *CqWUS1*/*CqWUS2* (Table 3, Figure 7). To evaluate the selection pressures on these duplicated gene pairs, the nonsynonymous (Ka) and synonymous (Ks) substitution rates, along with the Ka/Ks ratios, were calculated. The Ka/Ks values for all duplicated WOX gene pairs in quinoa were found to be less than one (Table 3), suggesting these genes experienced purifying selection. The Ka/Ks ratios for the segmentally duplicated *CqWOX* genes ranged from 0.04 to 0.21, with an average of 0.12. Additionally, an estimation of the divergence time for the *CqWOX* gene pairs indicated that duplication events took place between 6.52 and 14.62 MYA (Table 3).

**Table 3:** Nonsynonymous (Ka), synonymous (Ks), and Ka/Ks ratio between five duplicate *CqWOX* gene pairs.

**Gene 1**	**Gene 2**	**Ka**	**Ks**	**Ka/Ks**	**T** **(MYA)**	**Duplication type**
*CqWOX9*	*CqWOX8*	0.0085	0.0848	0.0998	6.5259	Segmental duplication
*CqWOX2*	*CqWOX3*	0.0090	0.1902	0.0475	14.6285	Segmental duplication
*CqWOX5*	*CqWOX6*	0.0097	0.1503	0.0643	11.5590	Segmental duplication
*CqWUS2*	*CqWUS1*	0.0271	0.1251	0.2169	9.6247	Segmental duplication
*CqWOX10*	*CqWOX7*	0.0315	0.1626	0.1936	12.5061	Segmental duplication

### 3.6. Detection of *cis*‐Elements in the Promoter Regions of *CqWOX*s

**Figure 8:**
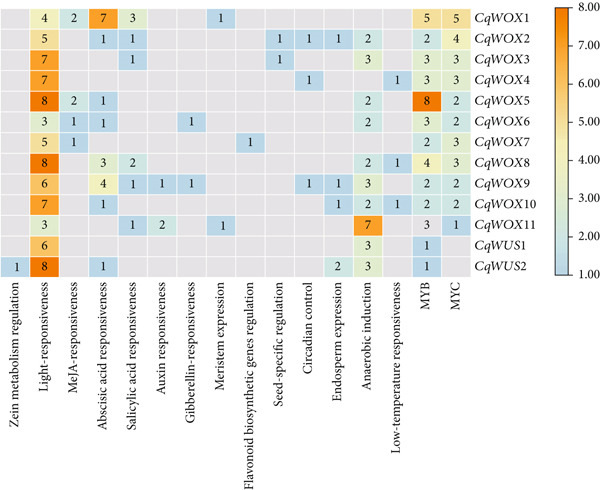
Promoter analysis of *CqWOX* genes. The numbers in the grids indicate the quantity of *cis*‐regulatory components associated with the indicated functions.

The PlantCARE database was utilized to examine the promoter sequences of 13 *CqWOX* genes to identify CREs. This assessment revealed 16 distinct types of CREs, classified into various functional categories (Figure 8). These elements were grouped into four main categories: hormone‐responsive, stress‐responsive, growth and development‐related, and light‐responsive elements. Core CREs such as the TATA‐box and CAAT‐box were excluded from this analysis, leading to the identification of numerous functional cis‐elements. Most of these elements were notably linked to light responsiveness, including GT1‐motif, G‐box, ATCT‐motif, GATA‐motif, and Box 4, which play significant roles in the transcriptional regulation mediated by light. Light‐responsive elements, in addition to MYB binding sites, were detected in all *CqWOX* genes. Moreover, MYC binding sites were found in 84.6% of the *CqWOX* genes; however, they were absent in *CqWUS1* and *CqWUS2* (Figure 8). Additionally, the results showed that *CqWOX5*, *CqWOX8*, and *CqWUS2* contained the uppermost number of light‐responsive elements, with *CqWOX5* displaying the most substantial MYB responsiveness. Interestingly, the *CqWOX9* and *CqWOX2* genes possessed 10 and 9 out of the 16 identified CRE types, respectively, demonstrating the highest diversity of CREs among the investigated genes (Figure 8). As illustrated in the figure, zein metabolism regulation (O_2_‐site) and flavonoid biosynthetic genes regulation (MBSI) were exclusively found in *CqWUS* and *CqWOX7*, respectively (Figure 8). These results shed light on the regulatory mechanisms controlling *CqWOX* gene expression and underscore their potential roles in light‐mediated transcriptional responses.

### 3.7. PPI Network of CqWOXs and GO

**Figure 9:**
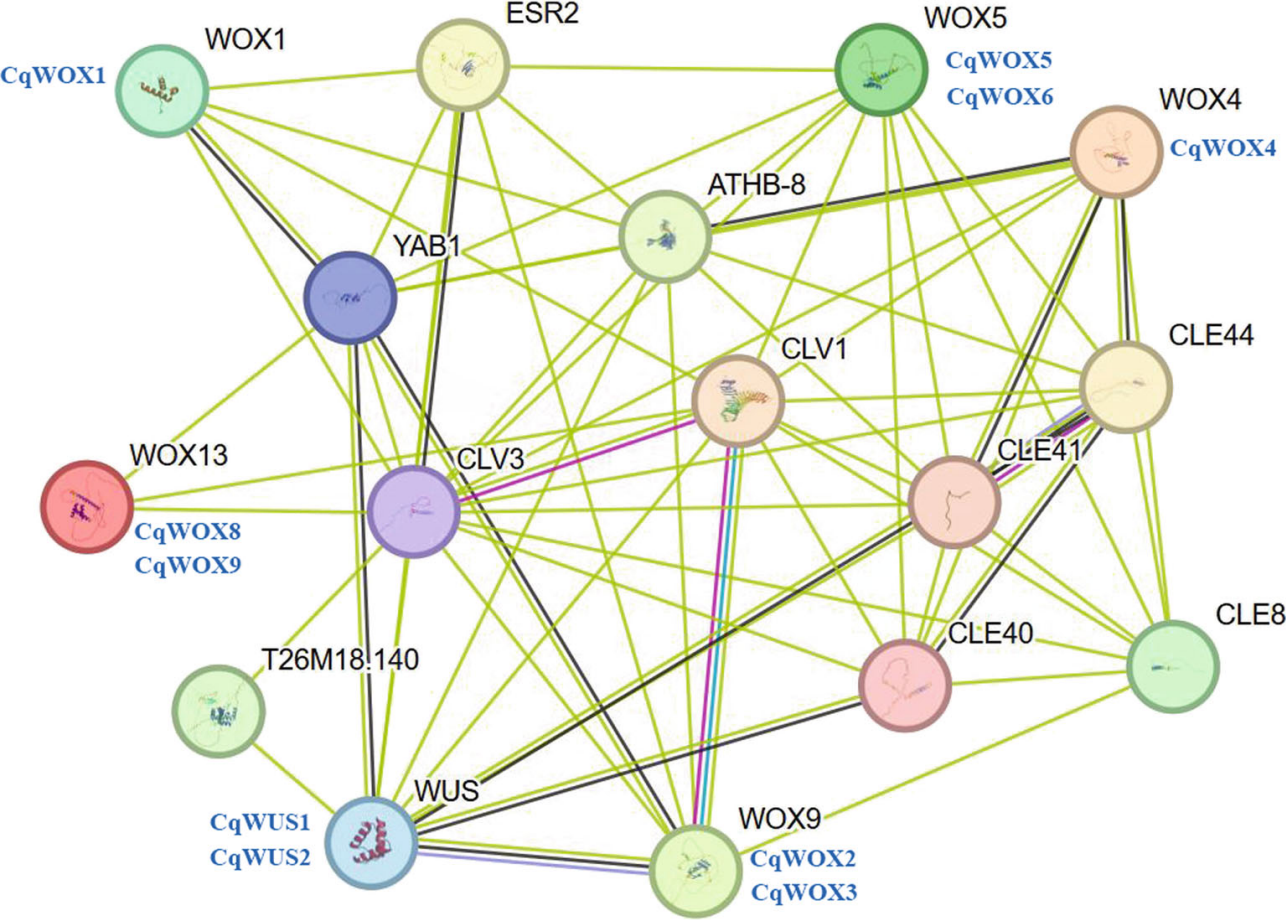
Protein–protein interaction network of CqWOX in Arabidopsis.

To investigate PPIs between CqWOX and other proteins in *C. quinoa*, an interaction protein network was created using the STRING web tools, drawing on Arabidopsis orthologs (Figure 9). This network encompassed 16 nodes, which included 6 Arabidopsis WOX proteins and 10 other proteins that displayed strong interactions with WOX proteins. The predicted network suggested the presence of CqWOX proteins within the established Arabidopsis PPI network, indicating that like interactions may occur in *C. quinoa*. Interestingly, the analysis showed that most of the Arabidopsis WOX proteins (four out of six) exhibited significant similarity with multiple CqWOX proteins, with each Arabidopsis WOX corresponding to up to two quinoa WOX proteins. This finding implies that certain members of CqWOX may possess conserved and synergistic regulatory functions similar to their Arabidopsis counterparts.

**Figure 10:**
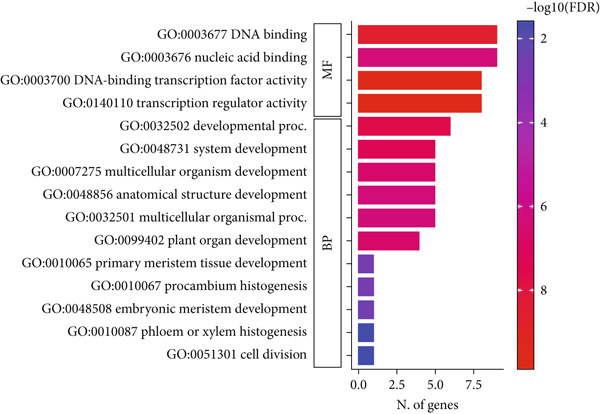
Gene Ontology (BP: biological process; MF: molecular function) enriched for *CqWOX* genes.

To illuminate the functional roles of *CqWOX* genes, a detailed GO analysis was conducted. The findings indicated that the *CqWOX* gene family is engaged in various regulatory functions across several GO categories, including molecular functions and biological processes (Figure 10). Within the biological process category, *CqWOX* genes are implicated in essential processes such as developmental processes (six genes), system development (five genes), multicellular organism development (five genes), and anatomical structure morphogenesis (five genes). Regarding molecular function, these genes are associated with nucleic acid binding (nine genes), DNA binding (nine genes), TF activity (eight genes), and transcription regulator activity (eight genes) (Figure 10).

### 3.8. RNA‐Seq Data Analysis

**Figure 11:**
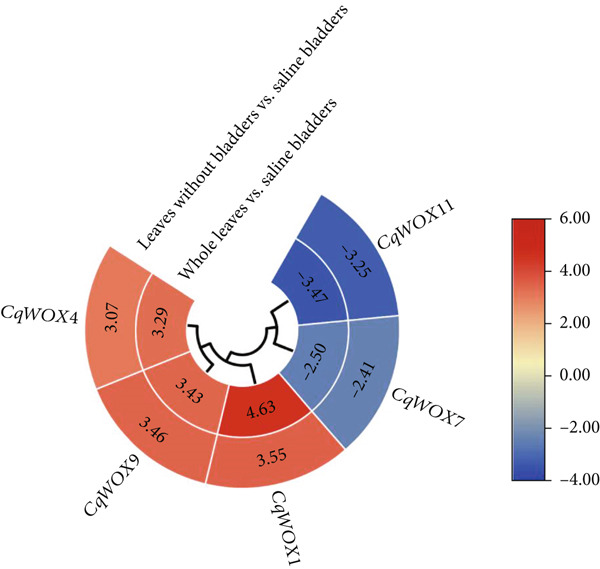
Heatmap illustration of expression profiles of differentially expressed *CqWOX* genes.

Transcriptomic analysis revealed that five members of the *CqWOX* gene family were differentially expressed in comparisons between whole leaves and saline bladders and between leaves without bladders and saline bladders. *CqWOX1*, *CqWOX4*, and *CqWOX9* exhibited significantly higher expression in leaves (fold change > 3), suggesting their repression in saline bladders. Conversely, *CqWOX7* and *CqWOX11* were significantly upregulated in saline bladders (fold change < −2), implying a potential role in the formation or function of these specialized structures (Figure 11). These differential expression patterns point to tissue‐specific functions of certain *WOX* genes, possibly related to salinity response or nodule regulation. Additionally, expression profiling under saline conditions in the stem and root tissues of the Q30 quinoa genotype showed upregulation of *CqWOX9* in the stem (fold change = 1.8) and downregulation of *CqWOX4* in the root (fold change = −1.7), further supporting the hypothesis that these genes have organ‐specific regulatory roles in response to salt stress.

## 4. Discussion

The number of WOX family members differs among plant species; nonetheless, they exhibit evolutionarily conservation and can be categorized into three distinct subgroups based on phylogenetic analysis [12]. As a vital gene family in plants, *WOX* genes code for proteins that govern cell division and differentiation, thus playing a critical role in plant growth and development [42]. These genes are involved in various developmental processes, including maintaining stem cell maintenance in meristems (WUS in the stem apical meristem, *WOX4* in the procambial meristem, and *WOX5* in the root apical meristem), embryo patterning [43], the development of lateral organs, and somatic embryogenesis [44]. Since the discovery of WUS in Arabidopsis, numerous *WOX* genes have been recognized and thoroughly researched in different crop plants, including wheat, maize, and canola [4, 45, 46]. In the current study, the WOX proteins of Arabidopsis were utilized as queries in a BLAST search, resulting in the identification of 13 *WOX* genes with homeodomain regions in quinoa (genome size 1.45 Gb). Phylogenetic analysis further classified these genes into three subgroups (Figure 2), a classification consistent with prior reports on other plant species. The number of *WOX* genes in quinoa was higher than that in some plant species like *H. annuus* (10 *HaWOX*s, genome size ~3.5 Gb) [15] and *C. sativus* (11 *CsWOX*s, genome size ~2248 Mb) [14], while being lower than that in other species including *A. thaliana* (15 *AtWOX*s, genome size 135 Mb) [13], *Brassica napus* (52 *BnWOX*s, genome size ~1.13 Gb), *Brassica rapa* (25 *BrWOX*s, genome size 455 Mb), *Brassica oleracea* (29 *BoWOX*s, genome size 660 Mb) [45], *Z. mays* (21 *ZmWOX*s, genome size 2.4 Gb) [4], and *Triticum aestivum* (43 *TaWOX*s, genome size ~17 Gb) [46]. This indicates that the number of *WOX* family genes does not correlate with the genome size of the respective plant species.

Proteins execute their functions within specific cellular environments determined by their subcellular localization. This localization is pivotal for regulating protein function by affecting the availability and accessibility of molecular interaction partners. Consequently, comprehending protein localization is essential for elucidating the cellular roles of both hypothetical and newly identified proteins [47]. In this study, subcellular localization investigation revealed that all CqWOX proteins, except for CqWOX2, were found within the nucleus (Table 1). To this point, experimental confirmation of subcellular localization has only been achieved for a portion of WOX family members. In *A thaliana*, WUS [48] and WOX6/PFS2 (PRETTY FEW SEEDS 2) [49] are recognized as nuclear‐localized, while WOX11 is also located in the nucleus in rice [50]. This localization within the nucleus may be facilitated by undetected cryptic nuclear localization signals (NLSs) by prediction tools or through associations with other proteins that possess NLS motifs. The presence of positively charged amino acid residues, arranged in compact sequences within the homeodomain, could potentially act as a cryptic NLS, facilitating nuclear targeting.

The calculation of the Ka/Ks ratio for the duplicated *CqWOX* genes indicates that these genes have experienced significant purifying selection, reflecting evolutionary constraints on their sequence and structure. The findings suggest that any mutations within these genes could be detrimental if they become fixed in the quinoa genome, likely because of their critical roles in the early development of embryos and flowers. The conservation of *CqWOX* genes underscores their functional significance in these developmental processes. Previous research in other species has shown that mutations affecting *WOX* family members can severely disrupt developmental processes. For instance, loss‐of‐function mutations have been reported to interfere with shoot and root apical meristem development [43] and negatively impact flower [8] and fruit development [51].

In addition to the homeodomain, certain WOX proteins have three additional functional domains: the acidic region (which is abundant in glutamic and aspartic acids), the WUS‐box (T‐L‐X‐L‐F‐P‐X‐X, where X represents an unspecified amino acid), and the EAR‐like motif (X‐L‐X‐L‐X‐L, where X denotes an uncertain amino acid). The WUS‐box is crucial for regulating stem cell identity and influencing the size of the floral meristem, while the acidic region acts as the primary activation domain for WUS proteins. In conjunction with the WUS‐box, the EAR‐like motif serves as a repression domain [40]. In this study, the WUS‐box (TLXLFP) was found in particular members of the WUS subgroup, including CqWOX1, CqWOX4–CqWOX6, CqWUS1, and CqWUS2. Furthermore, the EAR‐like motif (LXLXL, Motif 8) was determined to be specific to putative WUS genes (Figure 4). By definition, the WUS‐box motif is exclusively found in WUS subgroup members and is located at the carboxy‐terminal end of the homeodomain. Functional analyses have shown that the WUS‐box is vital for WUS activity, especially in regulating the stem cell population and floral patterning [40]. It is noteworthy that WUS subgroup members have a conserved two‐amino‐acid T‐L motif at the beginning of the WUS‐box, while non‐WUS WOX family members show variations at this position.

Sequence comparison revealed a high degree of similarity in the amino acid composition within the conserved homeodomain region of the 13 CqWOX proteins (Figure 5). Previous studies have identified 11 conserved amino acids in the homeodomain, including Q, Y, and E in Helix 1 and N, V, F, Y, K, F, Q, and R in Helix 3 [4, 52]. These residues were also conserved across most CqWOX proteins. In addition, conserved residues identified in Helix 2 included P, I, and L [52]. The secondary structure of the WOX proteins in quinoa was predicted using SOPMA, indicating the presence of four structural elements: alpha helices, extended strands, beta turns, and random coils. Among these, random coils were predominant, comprising more than 60% of the structure in all proteins, whereas beta turns were the least represented, accounting for only 1%–6% (Table 2), which was consistent with reports by Ramkumar et al. [53] in the *Apostasia shenzhenica*.

The *WOX* TF family is critical for stress tolerance, the regulation of plant growth and development, and the transduction of plant hormone signals [4, 28, 29]. The promoter of *CqWOX* genes includes *cis*‐acting elements linked to plant growth, development, hormonal regulation, and responses to stress. Previous research has shown that *WOX* genes are influenced by phytohormones such as indole‐3‐acetic acid (IAA) and abscisic acid (ABA) in the management of plant growth and development [54, 55]. In *A. thaliana*, WUS has been demonstrated to have a restrictive effect on the IAA signaling pathway, thereby preserving stem cell identity [56]. Moreover, various hormone‐responsive elements have been discovered within the *CqWOX* promoter region, including ABRE elements, auxin‐responsive elements, TCA elements, TGA elements, and GARE motifs. These results indicate that the *CqWOX* gene family plays a pivotal role in quinoa’s growth, development, and stress adaptation by mediating processes regulated by hormones.

The examination of the promoter regions of 13 *CqWOX* genes in quinoa has underscored the significance of light‐responsive elements, along with MYB and MYC binding sites, which play crucial roles in regulating plant development and responses to stress. The detection of multiple light‐responsive elements, including the GT1‐motif, G‐box, ATCT‐motif, GATA‐motif, and Box 4, in all *CqWOX* genes highlights the critical function of light in regulating these genes. These elements support transcriptional reactions to light, affecting aspects like photomorphogenesis and photosynthesis [57]. For instance, the G‐box is associated with the management of light‐responsive and stress‐related genes in Arabidopsis [58]. This indicates that *CqWOX* genes are effectively adapted to different light environments, fostering optimal growth and development.

The widespread occurrence of MYB binding sites in all *CqWOX* genes highlights their important function in stress responses. MYB TFs play a critical role in regulating a variety of abiotic stress responses, including drought and salinity [39]. The existence of MBS elements—MYB binding sites linked to drought responsiveness—in the promoter regions of these genes indicates a potential mechanism through which quinoa plants can adjust gene expression in order to adapt to challenging environmental conditions [17]. This adaptability is essential for survival in changing environments. Previous research on *WOX* genes has mainly focused on their functions in plant development, with few investigations into their roles in stress responses triggered by environmental changes. For instance, in *Camellia sinensis* (tea plant), *CsWOX13*, *CsWOX15*, and *CsWOX16* are significantly upregulated in response to phytohormone treatments and abiotic stresses [59]. Similarly, in *Populus × xiaohei*, the expression of *PsnWOX13c* and *PsnWOX13b* in roots is modulated under drought stress [60].

Additionally, the identification of MYC binding sites in 84.6% of the *CqWOX* genes indicates their involvement in integrating light signaling with other physiological processes. This relationship underscores a complex regulatory framework where MYC TFs influence light‐responsive gene expression, influencing plant development and adaptation to light conditions [57, 61]. In conclusion, the comprehensive analysis of CREs in *CqWOX* genes reveals an intricate regulatory landscape where light‐responsive elements, along with MYB and MYC binding sites, work together to finely tune gene expression in response to environmental cues. Understanding these interactions provides valuable insights into the molecular mechanisms that facilitate plant adaptability and resilience.

The PPI network constructed in this study included 16 nodes, comprising 6 Arabidopsis WOX proteins and 10 additional proteins (ESR2, T26M18.140, ATHB‐8, CLE44, CLE8, *CLAVATA1* [CLV1], *CLAVATA3* [CLV3], CLE40, CLE41, and YAB1) that have strong interactions with WOX proteins (Figure 9). A well‐known negative feedback loop exists between *WUS* as a first gene identified within the WOX family [62] and *CLV3*, which is essential for maintaining stem cell homeostasis in the shoot apical meristem (SAM) [63]. Previous research has indicated that WUS protein regulates *CLV3* expression within the organizing center by forming homodimers with itself and heterodimers with shoot meristemless. In response, *CLV3* interacts with *CLV1* and *CLAVATA2* (*CLV2*) to modulate *WUS* expression in the organizing center, thereby ensuring proper meristem function [64, 65]. Additionally, studies have shown that *WOX* genes play a significant role in the regulation of flowering and overall plant development [66].

*WOX5* is essential for keeping the columella stem cells (CSCs) in an undifferentiated condition, thus ensuring a steady generation of columella cells. A feedback loop exists in which differentiated CSCs produce the peptide CLAVATA3/ESR‐RELATED40 (CLE40), which interacts with receptor kinases ARABIDOPSIS CRINKLY4 (ACR4) and CLV1 to regulate the expression of *WOX5* [67]. Additionally, in Arabidopsis, secondary growth is driven by the meristematic activity of the vascular cambium. The TF *WOX4* is crucial for managing cell divisions within the cambium and serves as a significant target of the CLV3/EMBRYO SURROUNDING REGION‐RELATED 41 (CLE41) signaling pathway [68]. This emphasizes the important role of *WOX* genes in regulating both primary and secondary growth in plants.

Two key genes, *YAB1* and *CLV3*, are integral components of the CLAVATA (CLV)–WUS signaling pathway, which regulates stem cell maintenance in both shoot and floral meristems [69, 70]. *CLV3* is expressed in the central domain of the SAM and plays a crucial role in determining the relative positioning of organ primordia. It functions in conjunction with CLV1 and WUS to maintain the balance between stem cell proliferation and differentiation [71]. Meanwhile, YAB1 delineates the primordia domain at the periphery of the SAM and ensures robust partitioning of the meristem. It communicates nonautonomously with the central meristem to regulate *CLV3* and *WUS* expression, thereby coordinating organized SAM growth [72, 73]. This complex regulatory network underscores the critical role of *WOX* genes and their interactions with the CLV pathway in maintaining stem cell populations and ensuring proper meristem function in plant development.

The results of the GO analysis provide significant insights into the functional roles of *CqWOX* genes in several biological processes and molecular functions. Within the biological process category, it was found that *CqWOX* genes are linked to developmental processes, system development, multicellular organism development, and anatomical structure morphogenesis. These processes are crucial for proper growth and differentiation of plants, as *WOX* genes are well known for their involvement in maintaining meristems and forming organs. Research conducted on *A. thaliana* and *Z. mays* has shown that *WOX* genes are essential for embryogenesis, lateral organ formation, and vascular development [4, 74]. The involvement of *CqWOX* genes in these biological processes suggests their conserved role in plant developmental regulation, further supporting the hypothesis that they contribute to cellular differentiation. The molecular function analysis revealed that *CqWOX* genes are primarily involved in DNA binding, nucleic acid binding, DNA‐binding TF activity, and transcription regulator activity. These findings align with previous studies indicating that WOX proteins function as TFs regulating gene expression during key developmental processes [74–76]. In conclusion, the GO analysis provides strong evidence that *CqWOX* genes are essential for plant development and transcriptional regulation.

Transcriptomic analysis of quinoa identified tissue‐specific expression patterns for five *CqWOX* genes. Among these, *CqWOX1*, *CqWOX4*, and *CqWOX9* were repressed in saline bladders, whereas *CqWOX7* and *CqWOX11* were upregulated, suggesting their potential roles in bladder‐specific functions. Under salt stress, expression profiling revealed organ‐specific regulation, including the upregulation of *CqWOX9* in leaves and downregulation of *CqWOX4* in roots. These results imply that *CqWOX* genes may be involved in both salinity responses and the development of specialized tissues. In quinoa leaves, *CqWOX1* (an ortholog of *AtWOX1*) and *CqWOX4* (an ortholog of *AtWOX4*) exhibited the highest expression levels (Figure 11). In Arabidopsis, *AtWOX1* plays a critical role in leaf development, particularly in leaf blade expansion and margin formation, with single or double mutants displaying a narrow‐leaf phenotype [77]. *AtWOX4* shows dynamic expression in leaves, with promoter activity initially detected on the adaxial side of the lamina and later in the palisade parenchyma—though at reduced levels compared to the vasculature [78]. Additionally, *AtWOX4* is associated with vascular patterning and leaf complexity [79].

## 5. Conclusions and Future Perspective

In this study, we discovered 13 members of the *WOX* TF family in quinoa. The WUS subgroup, intermediate subgroup, and ancient subgroup are the three subgroups into which these genes were categorized according to evolutionary relationships. We also found five pairs of *WOX* genes that were segmentally duplicated in the quinoa genome. Purifying selection was applied to the genes, according to the Ka/Ks values. Furthermore, the *CqWOX* gene family had numerous hormone‐responsive, stress‐responsive, growth and development‐related, and light‐responsive components, according to the study of *cis*‐acting elements. A network of PPIs revealed that *WOX* genes are linked to the crucial genes *ESR2*, *T26M18.140*, *ATHB-8*, *CLE44*, *CLE8*, *YAB1*, *CLE40*, *CLE41*, *CLV1*, and *CLV3*. Overall, information about the general characteristics, evolutionary patterns, and functional variety of *CqWOX*s was made clear by the *WOX* genes found in the quinoa genome.

The study successfully identified the WOX gene family in quinoa through in silico analysis, complemented by RNA‐Seq data analysis, establishing a foundation for exploring their potential functions. Future research could focus on experimental validation methods, such as CRISPR/Cas9 gene editing, to better understand the regulatory networks involving *WOX* genes and their critical role in enhancing quinoa’s stress tolerance.

## Ethics Statement

The authors have nothing to declare.

## Conflicts of Interest

The authors declare no conflicts of interest.

## Author Contributions

B.B. designed the experiments, analyzed the data, and wrote the manuscript, and E.Z. analyzed the data. Each author actively contributed to the discussion, participated in writing, reviewed and approved the final published version, and is responsible for its content.

## Funding

This research did not receive any specific grant from funding agencies in the public, commercial, or not‐for‐profit sectors.

## Supporting information


**Supporting Information** Additional supporting information can be found online in the Supporting Information section. Supporting information is accessible online in the supporting description section. File S1: Detailed information on the synteny analysis of *CqWOX* genes. File S2: Detailed data on motif sequences of *CqWOX* genes identified in quinoa using MEME tools.

## Data Availability

The data that support the findings of this study are available from the corresponding author upon reasonable request.
