# Supplementary material for: WUSCHEL-Related Homeobox (WOX) Gene Family in Quinoa (Chenopodium quinoa): Genome‐Wide Identification and In Silico Characterization
Source: Int J Genomics. 2025 Sep 15;2025:7924847. doi: 10.1155/ijog/7924847 (PMC12434705; doi:10.1155/ijog/7924847)
Supplement: Supplementary file 1 — Supporting Information Additional supporting information can be found online in the Supporting Information section. Supporting information is accessible online in the supporting description section. File S1: Detailed information on the synteny analysis of CqWOX genes. File S2: Detailed data on motif sequences of CqWOX genes identified in quinoa using MEME tools. [file IJOG-2025-7924847-s001.zip › Supplementary file s1.docx]

| Table S1. Synteny Analysis of *CqWOX* genes | | | | | |
| --- | --- | --- | --- | --- | --- |
| Name | *Chenopodium quinoa* | *Arabidopsis thaliana* | *Amaranthus hypochondriacus* | *Beta vulgaris* | *Spinacia oleracea* |
| *CqWOX1* | AUR62017610 | Not detected | AH001120 | Bevul.9G017600.1 | Spov3_chr5.00846 |
| *CqWOX2* | AUR62014909 | AT2G33880.1  AT5G45980.1 | AH015282 | Bevul.8G017800.1 | Spov3_chr2.04548 |
| *CqWOX3* | AUR62031114 | AT5G45980.1 | AH015282 | Bevul.8G017800.1 | Spov3_chr2.04548 |
| *CqWOX5* | AUR62012213 | AT3G11260.1  AT5G05770.1 | AH016361 | Bevul.2G238700.1 | Spov3_chr4.03191 |
| *CqWOX6* | AUR62022846 | AT3G11260.1  AT5G05770.1 | AH016361 | Bevul.2G238700.1 | Spov3_chr4.03191 |
| *CqWOX7* | AUR62001809 | AT5G59340.1 | AH023456  AH013657 | Bevul.2G099500.1  Bevul.7G208600.1 | Spov3_chr2.02291 |
| *CqWOX8* | AUR62002048 | AT4G35550.1 | AH005965 | Bevul.6G074000.1 | Spov3_chr3.01312 |
| *CqWOX9* | AUR62003747 | AT4G35550.1 | AH005965 | Bevul.6G074000.1 | Spov3_chr3.01312 |
| *CqWOX10* | AUR62009597 | AT5G59340.1 | AH023456  AH013657 | Bevul.2G099500.1  Bevul.7G208600.1 | Spov3_chr2.02291 |
| *CqWOX11* | AUR62006532 | Not detected | AH011028 | Bevul.4G226100.1 | Spov3_chr1.00197 |
| *CqWUS1* | AUR62003373 | AT2G17950.1 | AH006421 | Bevul.6G035300.1 | Spov3_chr3.02325 |
| *CqWUS2* | AUR62029457 | AT2G17950.1 | AH006421 | Bevul.6G035300.1 | Spov3_chr3.02325 |
