# Supplementary material for: WUSCHEL-Related Homeobox (WOX) Gene Family in Quinoa (Chenopodium quinoa): Genome‐Wide Identification and In Silico Characterization
Source: Int J Genomics. 2025 Sep 15;2025:7924847. doi: 10.1155/ijog/7924847 (PMC12434705; doi:10.1155/ijog/7924847)
Supplement: Supplementary file 1 — Supporting Information Additional supporting information can be found online in the Supporting Information section. Supporting information is accessible online in the supporting description section. File S1: Detailed information on the synteny analysis of CqWOX genes. File S2: Detailed data on motif sequences of CqWOX genes identified in quinoa using MEME tools. [file IJOG-2025-7924847-s001.zip › Supplementary file s2.docx]

| Table S2. Motif sequences of *CqWOX* genes identified in quinoa using MEME tools. | | | | | |
| --- | --- | --- | --- | --- | --- |
| Motif ID | Motif Seq | Length | NSites | | E value |
| QLSEYGKIEGKNVFYWFQNHKARERQKQR | MEME-1 | 29 | 9 | 1.00E-127 | |
| RWNPTPEQIRILEEJYRSGGVTPPTDEII | MEME-2 | 29 | 13 | 2.90E-125 | |
| NPPPVTYSAPAVPTADSGNLKKILGGGEDMGEDGYASVNMENKHPELPIS | MEME-3 | 50 | 6 | 1.80E-38 | |
| MWAPEFTGVMRNVYGPVTCAKSIYEDDDGYMIVISLPFTDLQKVKVSWWN | MEME-4 | 50 | 2 | 9.30E-30 | |
| QTWNQEACDMLEVGLGTQCAKRLDSVWGAWFFFSFYFKPSLNEKSKSKIV | MEME-5 | 50 | 2 | 3.00E-25 | |
| VQHDMENMYMWVFKERPENALGKMQLRSYMNGHSRQGERPFPFSVEKGFV | MEME-6 | 50 | 2 | 5.00E-24 | |
| GDHDAPWTQLGDNLKPYAPAKYFGLJDKISCEPEDPYETLQ | MEME-7 | 41 | 4 | 1.10E-31 | |
| FPLNSYHEQESDKLRMFSNECCKENYATFAAYTFGRDHFDHPPLDLRLSF | MEME-8 | 50 | 2 | 2.50E-22 | |
| HKMQRKHYRGLSNPQCLHGIEIVRQPNLSAVDEEEQRRWMELTGRDLSF | MEME-9 | 49 | 2 | 1.50E-20 | |
| DSTDPNHHVSSLLDTSAAQSGKRDSVFASGGEEKSPEPK | MEME-10 | 39 | 6 | 2.20E-20 | |
| PFNVKEEFGEDAVLVHSGSGHPVMTNEWGVTVHPLQHGASYYLVGCVAA | MEME-11 | 49 | 2 | 4.20E-20 | |
| TACKPYIQRNDRTFKLTDPSPEHCPPGDFKREIPLPSRIPDDAKLEAYFD | MEME-12 | 50 | 2 | 8.00E-20 | |
| HHDDASATINVPASSDQAVGGFSSLLLGDMFGGDHSKRDHEVEKLKFQQE | MEME-13 | 50 | 2 | 4.00E-16 | |
| HLGFYESIMQNPRIDDLIGKIEVPGSYNSYQSTEDYNMTG | MEME-14 | 40 | 2 | 5.30E-14 | |
| YYTGGYRWSNGSGGNTSLELSLNSYGYGYYSPNI | MEME-15 | 34 | 2 | 9.10E-10 | |
